# Implementation of Isavuconazole in a Fluorescence-Based High-Performance Liquid Chromatography Kit Allowing Simultaneous Detection of All Four Currently Licensed Mold-Active Triazoles

**DOI:** 10.1128/mSphere.00098-17

**Published:** 2017-05-10

**Authors:** René Jørgensen, Siri Rytcher Andersen, Karen Marie Thyssen Astvad, Maiken Cavling Arendrup

**Affiliations:** a Unit of Mycology, Department of Microbiology and Infection Control, Statens Serum Institut, Copenhagen, Denmark; b Department of Clinical Microbiology, Rigshospitalet, Copenhagen, Denmark; c Department of Clinical Medicine, University of Copenhagen, Copenhagen, Denmark; Carnegie Mellon University

**Keywords:** HPLC, isavuzonazole, TDM, therapeutic drug monitoring, fluorescence detection, triazoles

## Abstract

Isavuconazole (ISZ) is a newly available broad-spectrum triazole agent recently approved for the treatment of both invasive aspergillosis and mucormycosis. The aim of this study was to develop a simple and reliable method for therapeutic drug monitoring (TDM) of ISZ in human plasma samples. The method involves using a kit from ChromSystems intended for TDM of itraconazole (ITZ), posaconazole (PSZ), and voriconazole (VRZ) in serum/plasma for sample preparation and high-performance liquid chromatography, using fluorescence detection with emission and excitation wavelengths set to 261 and 366 nm, respectively. The assay was linear over the ISZ concentration range of 0.2 to 20.0 mg/liter, using a 0.1-ml sample volume. The inter- and intraday coefficients of variation were all below 3.7%, whereas the accuracies ranged from 95.0 to 106.2% and the mean extraction recovery was 91.9%. In addition, the method worked well using four different Vacutainer types, with six different healthy volunteers and under a number of relevant storage conditions. Finally, the ISZ detection could be seamlessly implemented in the TDM kit for VRZ, PSZ, and ITZ, enabling simultaneous detection of all four triazoles. This method proved to be simple, accurate, precise, and well suited for routine analysis work. It has been implemented in our laboratory for the simultaneous quantitative analysis of ISZ, VRZ, PSZ, and ITZ for TDM and pharmacokinetic research.

**IMPORTANCE** Isavuconazole is a new broad-spectrum triazole agent recently approved for the treatment of both invasive aspergillosis and mucormycosis. Currently, there is no consensus regarding the potential need for TDM of isavuconazole, and no therapeutic window has been defined. However, at the ECIL-6 meeting in 2015, it was advised that TDM is indicated in a number of different settings. In this study, we describe a rapid and validated isocratic HPLC method for fluorescence-based detection and quantification of isavuconazole in human plasma/serum samples. The method is simple and efficient with good accuracy and precision and importantly only requires a small volume of patient plasma/serum. Furthermore, this method is highly sensitive and selective and can be detected simultaneously with the three other triazoles, itraconazole, voriconazole, and posaconazole, without the need for expensive mass spectrometry equipment.

## Abstract (precis)

Isavuconazole is a new broad-spectrum triazole agent recently approved for the treatment of both invasive aspergillosis and mucormycosis. Currently, there is no consensus regarding the potential need for TDM of isavuconazole, and no therapeutic window has been defined. However, at the ECIL-6 meeting in 2015, it was advised that TDM is indicated in a number of different settings. In this study, we describe a rapid and validated isocratic HPLC method for fluorescence-based detection and quantification of isavuconazole in human plasma/serum samples. The method is simple and efficient with good accuracy and precision and importantly only requires a small volume of patient plasma/serum. Furthermore, this method is highly sensitive and selective and can be detected simultaneously with the three other triazoles, itraconazole, voriconazole, and posaconazole, without the need for expensive mass spectrometry equipment.

## INTRODUCTION

Therapeutic drug monitoring (TDM) is indicated for serious infections when the therapeutic window is narrow or bioavailability is unreliable. In mycology, this is the case for flucytosine, itraconazole (ITZ), voriconazole (VRZ), and posaconazole (PSZ).

**FIG 1:**
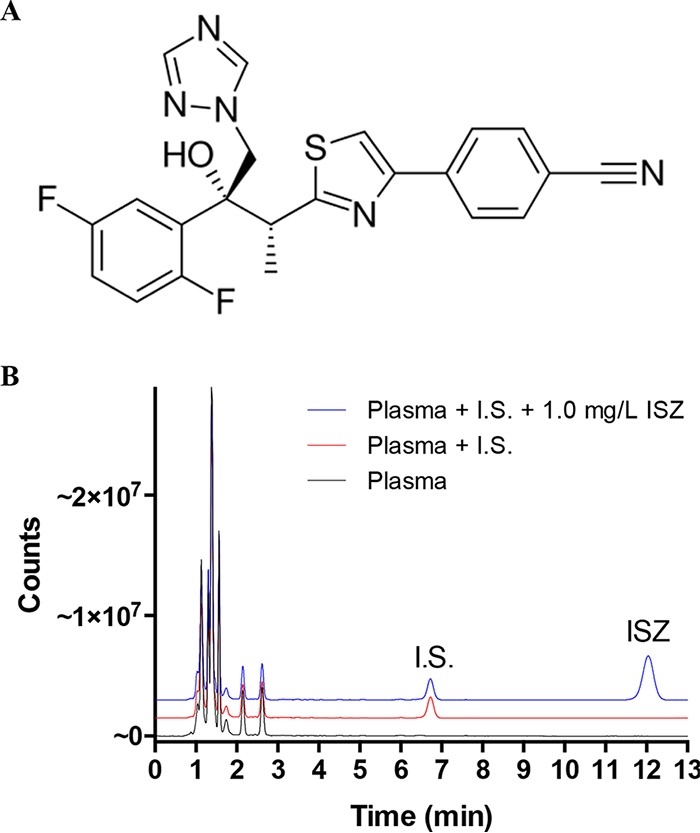
Detection of ISZ. (A) Chemical structure of ISZ. The chemical formula is C_22_H_17_F_2_N_5_OS, with a molecular mass of 437.47 g/mol. (B) Chromatogram on filtered pooled human plasma spiked with ISZ and prepared according to the instruction manual for the TDM kit. For improved visualization the red and blue lines were transformed by adding 1.5 and 3.0 million counts, respectively, to the measured values at all time points. A clear symmetric peak for ISZ appears in the chromatogram, with a retention time around 12.1 min. I.S., internal standard.

Isavuconazole (ISZ) (4-{2-[(1*R*,2*R*)-(2,5-difluorophenyl)-2-hydroxy-1-methyl-3-(1*H*-1,2,4-triazol-1-yl)-propyl]-1,3-thiazol-4-yl}benzonitrile) is a novel triazole with a broad-spectrum *in vitro* activity against a range of clinically relevant yeasts and molds (Fig. 1A) (1–4). It is available as a prodrug (isavuconazonium sulfate) which is rapidly and completely converted to the active form by plasma esterases. It is licensed for primary therapy of invasive aspergillosis in adults by both the U.S. Food and Drug Administration (FDA) and the European Medicines Agency (EMA) and for primary (FDA) and second-line (EMA) therapy for invasive mucormycosis. Standard dosing is 200 mg three times daily the first 2 days (a total of 6 times), then 200 mg daily (intravenous, parenteral, or oral therapy), with no licensed dose escalation option (5, 6). The bioavailability is 98% and is not affected by food intake or gastric pH. Isavuconazole is extensively distributed, with a mean steady-state volume of distribution of approximately 450 liters. It is highly bound (>99%) to human plasma proteins, predominantly to albumin. *In vitro*/*in vivo* studies indicate that the cytochrome P450 enzymes CYP3A4, CYP3A5, and subsequently UDP glucuronosyltransferases (UGT) are involved in the metabolism of ISZ (5).

In a randomized, controlled, noninferiority phase III clinical trial comparing ISZ to VRZ for primary treatment of invasive mold disease caused by *Aspergillus* spp. and other filamentous fungi, the steady-state ISZ trough plasma concentration ranged from 0.81 to 9.95 mg/liter, with a mean of 3.4 mg/liter (standard deviation [SD], 1.8 mg/liter) (7). The EUCAST epidemiological cutoff value for *Aspergillus fumigatus* is 2 mg/liter. However, the ISZ MICs for wild-type *A. fumigatus* isolates and for *A. fumigatus* isolates harboring *CYP51A* azole target gene mutations overlap. Therefore, a MIC of 2 mg/liter may represent a wild-type isolate, or a mutant isolate with reduced susceptibility. Monte Carlo simulations using the data from the clinical trial suggested that for patients with infections due to isolates with an MIC of 2 mg/liter, approx. 25% will not achieve an area under the concentration-time curve (AUC)/MIC ratio of 33, which is needed for 90% efficacy (8).

Currently, there is no consensus regarding the potential need for TDM for ISZ, and no therapeutic window has been defined. At the ECIL-6 meeting in 2015, it was advised that TDM is indicated in the setting of breakthrough or infections unresponsive to treatment, treatment of pathogens with reduced susceptibility, or in the setting of drug interactions (CIII recommendation), but it was also clearly highlighted that additional data are needed (9). Currently, there are only a few published validated detection methods for TDM on plasma/serum samples that include ISZ. One publication involved ultraperformance liquid chromatography (UPLC) with UV detection (10), while two other publications included detection by tandem mass spectrometry (11, 12).

We routinely perform simultaneous TDM of ITZ, VRZ, or PSZ. For this purpose, we use a fluorescence-based HPLC TDM kit commercially available from ChromSystems. This method is quick, reliable, and highly selective. Also, importantly, this method only relies on an isocratic mobile phase using a simple HPLC system equipped with a fluorescence detector. To our knowledge, there is no published description of a validated method for fluorescence-based detection of ISZ in human plasma or serum. Since ISZ is structurally related to the three other triazoles, we decided to determine if the same fluorescence-based HPLC TDM kit could also be used for TDM of this new antifungal.

## RESULTS AND DISCUSSION

### Isavuconazole structure and chromatographic characteristics.

Because of its fluorescent properties, ISZ can be detected using a fluorescence detector with emission and excitation wavelengths set to 261 and 366 nm, respectively. To perform the plasma sample preparation, we used the reagents included in the HPLC-based TDM kit from ChromSystems that is intended for simultaneous detection of ITZ, PSZ, and VRZ. This kit also includes an acetonitrile-based mobile phase for the HPLC. Figure 1B shows an analytical chromatogram for blank filtered pooled human plasma, a quality control (QC) sample including an internal standard, and a sample spiked with ISZ at 1.0 mg/liter. All three samples were run for 13 min, as suggested in the ChromSystems manual. The retention time of ISZ was approximately 12.1 min, and the peak was well eluted by the end of the 13-min run time cycle.

### Calibration curve.

**FIG 2:**
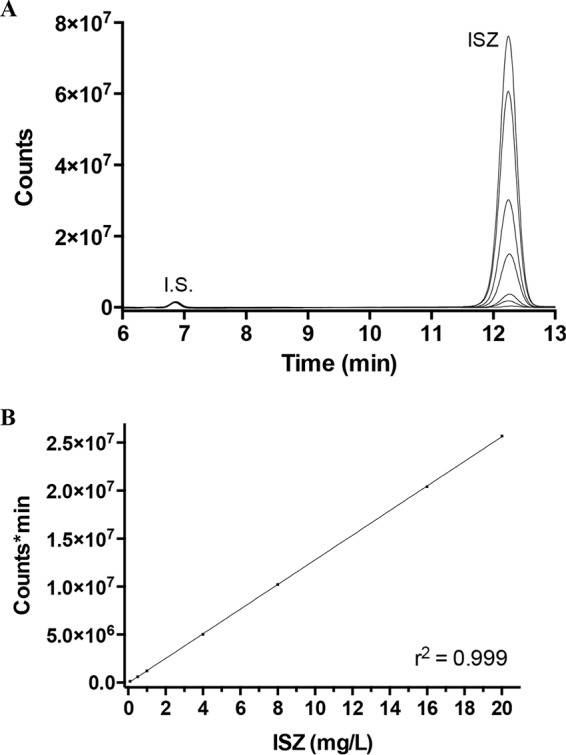
Calibration of ISZ. (A) Chromatogram of prepared ISZ-spiked human plasma samples at different concentrations, from 0.2 to 20 mg/liter. I.S., internal standard. (B) A calibration curve based on the integrated area of the ISZ peaks, averaged from two repetitions, shows that the concentration range is linear. The *r*^2^ value is the result of a linear fit, calculated by using Prism 6.

To create a calibration curve, human plasma samples were spiked with ISZ at seven different concentrations ranging from 0.2 to 20.0 mg/liter and applied to the HPLC column (Fig. 2A). Plotting the integrated areas as a function of concentration revealed that the assay was linear within the range and resulted in a correlation coefficient (*r*^2^ > 0.99) with regression intercepts not statistically different from zero (Fig. 2B). At ISZ concentrations higher than 20 mg/liter, the fluorescence detector quickly reached the saturation level, and hence this concentration was set as the upper limit of quantification.

### Lower limit of quantification.

The lower limit of quantification (LLOQ) was determined to be 0.2 mg/liter for ISZ in filtered pooled human plasma, as lower concentrations resulted in unacceptable accuracy and precision values.

### Accuracy, precision, and recovery.

The method showed good accuracy and precision in the filtered pooled human plasma samples. Table 1 summarizes the intra- and interday accuracy and precision values for ISZ in plasma samples. The average intraday and interday values for LLOQ and the three QC samples were 99.4% and 101.1%, respectively. Similarly, average intraday and interday precision coefficients of variation (CVs) were 2.30% and 2.74%, respectively. Hence, the sensitivity of the method was satisfactory and sufficiently covered the therapeutic range of ISZ in plasma, which can be expected to lie approximately between 0.5 and 10.0 mg/liter (7). Finally, Table 1 also shows that the extraction mean recoveries of ISZ in the spiked plasma samples ranged from 84.5% to 101.8%, with an average of 91.9%. No detectable carryover was observed in a number of runs where a blank sample was injected immediately after running several high-concentration quality control (HQC) samples on the HPLC column.

**TABLE 1:** Intraday precision, accuracy, and average recovery and interday precision and accuracy for ISZ in filtered pooled human plasma samples^a^

Comparison and added concn (mg/liter)	Found concn (mg/liter [mean ± SD])	Accuracy^b^ (%)	Precision^c^ (% RSD)	Recovery^d^ (mean % ± SD)
Intraday				
LLOQ (0.2)	0.190 ± 0.004	95.0	2.07	84.5 ± 3.3
LQC (0.3)	0.311 ± 0.003	103.8	0.86	88.5 ± 1.9
MQC (8.0)	7.908 ± 0.273	98.8	3.45	92.6 ± 2.6
HQC (20.0)	20.008 ± 0.558	100.0	2.79	101.8 ± 4.4
All samples		99.4 ± 3.6	2.30 ± 1.1	91.9 ± 7.4
Interday				
LLOQ (0.1)	0.212 ± 0.003	106.2	1.60	
LQC (0.3)	0.296 ± 0.007	98.6	2.42	
MQC (8.0)	8.004 ± 0.261	100.0	3.26	
HQC (20.0)	19.910 ± 0.734	99.5	3.69	
All		101.1 ± 3.4	2.74 ± 0.9	

a Intraday precision, accuracy, and average recovery were measured within a single day (*n* = 5), and interday precision and accuracy were measured on four separate days (*n* = 20). Five individually prepared samples were evaluated at each concentration level.

b Accuracy is the percentage of the nominal value.

c Precision is expressed as the percentage of the RSD of each calculated concentration.

d Recovery is the percent extraction mean recovery.

### Matrix effect and selectivity.

Intraday accuracy and precision values on blood collected from the same person in four different BD Vacutainer tubes (including a tube for serum separation) revealed no significant matrix effects (Table 2). Furthermore, the selectivity of the method was evaluated by analyzing serum from six volunteers; in these samples no interference by endogenous components was noted (see Fig. S1 in the supplemental material). In particular, we saw no interference around the ISZ retention time and intraday accuracy, and precision values on low-concentration quality control (LQC) and HQC serum samples from the volunteers were also well within the limits of the EMA guidelines (Table S1). Also, according to the instruction manual of the TDM kit, several known compounds can cause additional peaks in the chromatogram. However, these compounds primarily elute with retention times between 3.2 and 7.8 min. In addition, the instruction manual reports a broad range of compounds which have been tested and shown to not interfere with this kit’s performance.

**TABLE 2:** Matrix effect of ISZ

Vacutainer tube and sample concn^a^ (mg/liter)	Found concn (mean mg/liter ± SD)	Accuracy^b^ (%)	Precision^c^ (% RSD)
CSC			
LQC (0.3)	0.307 ± 0.008	102.3	2.50
HQC (20.0)	19.599 ± 0.391	98.0	2.00
K2E			
LQC (0.3)	0.309 ± 0.012	103.0	4.04
HQC (20.0)	19.901 ± 0.368	99.5	1.85
LH			
LQC (0.3)	0.299 ± 0.006	99.5	2.04
HQC (20.0)	20.271 ± 0.141	101.4	0.70
Serum			
LQC (0.3)	0.316 ± 0.020	105.3	6.18
HQC (20.0)	19.238 ± 0.636	96.2	3.31

a Intraday precision and accuracy values were determined for the LQC and HQC in human blood samples collected in four different BD Vacutainer tubes. There were five individually prepared samples at each concentration level. CSC, coagulation sodium citrate (3.2%); K2E, EDTA; LH, lithium heparin; serum, clot activator for serum separation.

b Accuracy is the percentage of the nominal value.

c Precision is expressed as the percentage of the RSD of each calculated concentration.

10.1128/mSphere.00098-17.1 FIG S1  Chromatogram of serum samples spiked with ISZ from six healthy volunteers (A to F). The concentration of ISZ was 0.3 mg/liter, and it was prepared together with the internal standard (I.S.) supplied with the TDM kit. Download FIG S1, PDF file, 0.1 MB. Copyright © 2017 Jørgensen et al. 2017 Jørgensen et al. This content is distributed under the terms of the Creative Commons Attribution 4.0 International license.

10.1128/mSphere.00098-17.2 TABLE S1  Measured concentrations of ISZ in serum from healthy volunteers (intraday precision and accuracy values were determined for the LQC and HQC in serum samples collected from six different volunteers; five individual samples were prepared at each concentration level). Download TABLE S1, PDF file, 0.1 MB. Copyright © 2017 Jørgensen et al. 2017 Jørgensen et al. This content is distributed under the terms of the Creative Commons Attribution 4.0 International license.

### Stability.

The influence of various possible storage conditions between sample collection and analysis were examined using two concentrations of ISZ (LQC and HQC). Working solutions of ISZ dissolved in filtered pooled human plasma showed no perceptible degradation between solutions kept at room temperature for 24 h versus 7 days, at −20°C for 48 h versus 60 days, or in freshly prepared samples (Table 3). Also, three freeze-thaw cycles of working solutions indicated that ISZ was stable under these conditions. Finally, we found that working solutions of ISZ mixed with the internal standard and stored for 24 h at room temperature, as well as prepared samples stored for 24 h in the autosampler at room temperature, were stable.

**TABLE 3:** Stability of ISZ in plasma^a^

Storage condition and sample concn (mg/liter)	Found concn (mean mg/liter ± SD)	Accuracy^b^ (%)	Precision^c^ (% RSD)
24 h at 20°C			
LQC (0.3)	0.300 ± 0.003	100.1	0.833
HQC (20.0)	19.603 ± 0.142	98.0	0.727
7 days at 20°C			
LQC (0.3)	0.292 ± 0.005	97.2	1.652
HQC (20.0)	19.373 ± 0.420	96.9	2.170
Freeze (−20°C) for 48 h			
LQC (0.3)	0.312 ± 0.008	104.0	2.606
HQC (20.0)	20.265 ± 0.575	101.3	2.840
Freeze (−20°C) for 60 days			
LQC (0.3)	0.319 ± 0.040	106.4	12.545
HQC (20.0)	22.119 ± 0.941	110.6	4.255
Three freeze-thaw cycles			
LQC (0.3)	0.308 ± 0.014	102.7	4.611
HQC (20.0)	21.526 ± 0.500	107.6	2.321
Sample + IS for 24 h at 20°C			
LQC (0.3)	0.298 ± 0.009	99.2	3.082
HQC (20.0)	21.654 ± 0.275	108.3	1.272
In autosampler for 24 h at 20°C			
LQC (0.3)	0.291 ± 0.005	97.0	1.598
HQC (20.0)	19.172 ± 0.542	95.9	2.825

a Intraday precision and accuracy values were determined for the LQC and HQC in filtered pooled human plasma samples after storage under various conditions. Five individually prepared samples were used for each concentration level and storage condition. IS, internal standard.

b Accuracy is the percentage of the nominal value.

c Precision is expressed as the percentage of the RSD of each calculated concentration.

### Simultaneous detection of ISZ, VRZ, PSZ, and ITZ.

**FIG 3:**
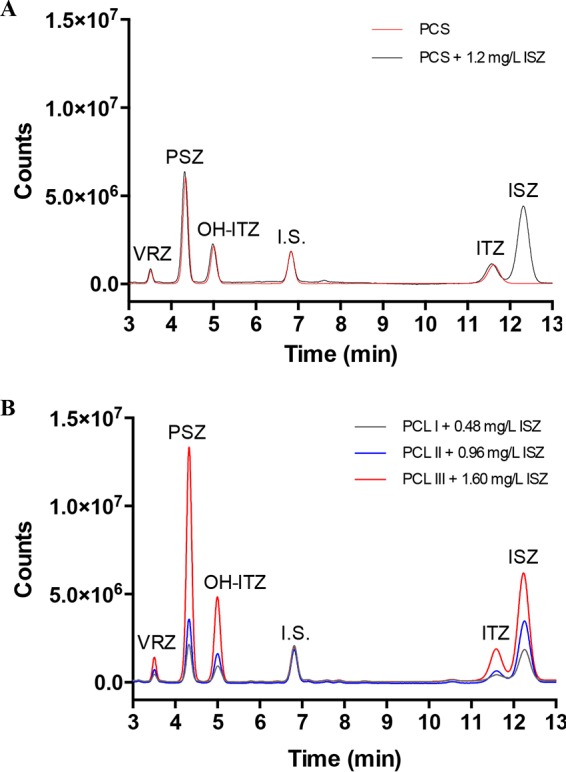
Simultaneous detection of ISZ together with ITZ, OH-ITZ, PSZ, and VRZ. (A) Chromatogram of the PCS control before and after reconstituting the lyophilized mix of compounds with plasma containing 1.2 mg/liter of ISZ. I.S., internal standard. (B) Chromatogram of PCL I, II, and III calibrators after reconstituting the lyophilized mix of compounds with plasma containing 0.48, 0.96, and 1.60 mg/liter of ISZ, respectively.

As ISZ can be detected using the TDM kit, we decided to attempt simultaneous detection of all four mold-active triazoles. Initially, we added 1 ml of human plasma containing 1.2 mg/liter ISZ to a vial of the plasma calibration standard (PCS) containing lyophilized VRZ, PSZ, ITZ, OH-ITZ, and the internal standard. This was then used for sample preparation, applied to the HPLC column, and compared with a PCS without ISZ (Fig. 3A). The chromatogram revealed that ISZ is generally well separated from the other analytes and that adding ISZ did not interfere with any of the other integrated peak areas. For simultaneous creation of calibration standards, we also added various concentrations of ISZ to the three plasma control level vials (PCL I to III) as described above (Fig. 3B). Again, ISZ was well separated from the other analytes, although at higher concentrations of the analytes (PCL III) the peaks of ITZ and ISZ slightly overlapped. However, from the three chromatograms, reliable linear calibration curves of all the analytes could be generated (data not shown).

### Conclusions.

This is the first report of a rapid and validated isocratic HPLC method that describes the fluorescence-based detection and quantification of ISZ in human plasma samples. We showed that ISZ is linear within a therapeutic concentration range of 0.2 to 20.0 mg/liter. The ISZ retention time of approximately 12.1 min allows the run time to remain within the recommended 13 min according to the instruction manual for the kit. The method is simple and efficient, with good accuracy and precision, and importantly, it only requires a small volume of human plasma/serum. Fluorescence-based detection is generally more sensitive and selective than UV absorption and offers high reliability in the identification and determination of compounds of interest while still avoiding the requirement for expensive mass spectrometry equipment. Hence, this method is suitable for the estimation of ISZ at different therapeutic dose levels for pharmacokinetic studies as well as TDM in adults and children. However, during the validation of this method, we occasionally experienced that the standard deviation of the internal standard measurements in a sequence of samples were higher than the ISZ measurements, and at times this resulted in elevated accuracy and precision values. This phenomenon is also seen when using the kit purely for detection of ITZ, PSZ, or VRZ, but it only rarely calls for repetition of the experiment. It is possible that using a different kind of internal standard could optimize the method. The extraction mean recoveries of ISZ-spiked plasma samples (LLOQ and the three QCs) compared to direct injections of ISZ dissolved in the mobile phase were all acceptable. However, the recovery values showed a trend toward lower recovery at lower ISZ concentrations. Possibly, the ISZ-binding capability of the plasma proteins causes the removal of small amounts of ISZ when the protein is precipitated during sample preparation.

Additionally, a major advantage of this methodology is that the kit allows for simultaneous quantification of ISZ together with the three other triazoles. This is convenient and clinically advantageous. First, as neither column nor mobile phase needs to be changed depending on the specific azole concentration requested, a short turnover time is allowed. Second, a relevant result is obtained without delay even in the scenario where an incorrect request has been placed, which in our experience is not a rare event (e.g., due to change of medication but not sampling order). When larger concentrations of ITZ and ISZ are both present in the sample, the two peaks are not separated completely from each other. However, this is a rare phenomenon since the two compounds are not given in combination and given the fact that it is recommended to measure drug levels at steady state (day 4 to 7), meaning that in the scenario of a switch from ITZ to ISZ the level of ITZ will inevitably be low due to timing and often poor availability when it is indicated to measure ISZ. In the event of switching from ISZ to ITZ, it cannot be excluded that significant amounts might be present at the time of clinically indicated ITZ TDM. In such cases, we advise to use the heights of the two peaks when the areas overlap. We see no difference in accuracy and precision when measuring peak heights compared to measuring peak areas.

## MATERIALS AND METHODS

### Chemicals and standards.

Isavuconazole was kindly supplied by Basilea Pharmaceutica Ltd. (Basel, Switzerland). The standard stock solution of ISZ was prepared at a concentration of 5.000 mg/liter in dimethyl sulfoxide (≥99.7% pure; Chromasolv Plus) and diluted to a 160-mg/liter working solution in filtered pooled human plasma (BioWest) or plasma from healthy volunteers. The stock solution and working solution were stored at −20°C. The chemicals for sample preparation and HPLC were obtained from the itraconazole/posaconazole/voriconazole reagent kit for TDM on plasma/serum samples available in Europe (ChromSystems Instruments and Chemicals GmbH, Germany), which also included a plasma calibration standard (PCS), plasma control level I, II, and III (PCL I to III), and also the HPLC column provided for the kit. The PCS is a control designed for calibrating the measurement method and contains predetermined concentrations of ITZ (0.83 mg/liter), OH-ITZ (1.1 mg/liter), VRZ (2.95 mg/liter), PSZ (2.38 mg/liter), and internal standard. A PCS including ISZ was prepared by diluting the ISZ working solution to 1.2 mg/liter in filtered pooled human plasma before using 1 ml of this solution to reconstitute the PCS according to the instructions of the manufacturer. PCL I, II, and III are designed for calibrating the measurement method and contain the following predetermined concentrations of ITZ (0.28, 0.56, and 1.37 mg/liter), OH-ITZ (0.42, 0.83, and 2.3 mg/liter), VRZ (1.01, 2.46, and 4.72 mg/liter), and PSZ (0.57, 1.19, and 4.72 mg/liter) for preparation of calibration curves. PCL I to III, including ISZ, were prepared by diluting the working solutions to 0.48, 0.96, and 1.60 mg/liter, respectively, in filtered pooled human plasma and using 1-ml volumes of these solutions for reconstituting PCL I, II, and III. It should be noted that according to the instructions of the manufacturer, distilled water should be used for the reconstitution of PCS and PCL I to III. However, we experienced no differences in concentration values whether we used water or human plasma (data not shown).

### Instruments and chromatographic conditions.

Analyses were carried out on a Vanquish UHPLC system (Thermo Fisher Scientific) consisting of a binary pump (VH-P1-A), an autosampler with thermostat and column compartment (VH-A10-A and VH-C10-A), and a fluorescence detector (VF-D50-A). The fluorometric detection was conducted using emission and excitation wavelengths set at 261 and 366 nm, respectively. The data were acquired and processed by using the Chromeleon 7.2 chromatography data system software. Chromatographic separation was carried out on an HPLC column (supplied for the kit by ChromSystems), with a column temperature of 25°C and an isocratic flow rate of the mobile phase (also supplied with the TDM kit from ChromSystems) of 1.2 ml/min. The run time for each sample was 13 min. All plots were prepared using Prism 6.0 (GraphPad Software, Inc.).

### Sample preparation.

The plasma samples were prepared according to the instruction manual for the TDM kit. Briefly, 100 μl of human plasma sample was mixed with 25 μl of internal standard and 25 μl of precipitation reagent I. After vortexing, 200 μl of precipitation reagent II was added and the mixture was vortexed for 30 s before centrifuging for 5 min at 15,000 × *g*. The supernatant was then transferred into an HPLC glass vial and placed in the sample chamber, followed by injection of 20 μl of sample into the HPLC system.

### Calibration.

Plasma samples spiked with ISZ were prepared in filtered pooled human plasma at the following seven concentrations: 0.2, 0.5, 1.0, 4.0, 8.0, 16.0, and 20.0 mg/liter. Each concentration was determined as the average of two injections on the HPLC column from dilutions prepared independently from the QC samples (see below). All calibration curves were constructed using the ratio of the observed peak area of ISZ and the internal standard, followed by linear regression analysis (least absolute deviation) of the data using the equation *y = mx + b*, where *y* was the peak ratio, *x* the concentration of ISZ, and *m* and *b* the slope and intercept of the curve, respectively (GNU octave 4.0.3, Prism 6; GraphPad Software, Inc.). A correlation coefficient (*r*^2^) of >0.99 was desirable for each calibration curve.

### LLOQ and QC samples.

The LLOQ is defined by the EMA guidelines for bioanalytical method validation (13) as being at least 5 times the signal of a blank sample and the minimum amount that gives precise measurements (accuracy and precision both within 20% of the nominal value). The low-concentration QC (LQC) was selected as being within three times the LLOQ, the medium QC (MQC) was between 30 and 50% of the calibration curve range, and the high QC (HQC) was at least 75% of the upper calibration curve range.

### Accuracy, precision, and recovery.

Accuracy and precision were determined as defined by the EMA guidelines (13). Accuracy is the mean of the measured LLOQ, LQC, MQC, and HQC concentrations relative to the theoretical value and is reported as a percentage of the nominal value. The overall mean precision describes the closeness of repeated measures of an analyte expressed as the CVs and is reported as the percent relative standard deviation (RSD) of each calculated concentration. Both precision and accuracy values were expected to be within ±15% of the nominal value for all concentrations except for the LLOQ, for which +20% was acceptable.

The intraday precision and accuracy values for plasma LLOC and QCs were calculated from five independently spiked preparations, as described above, with each measured in duplicate on the same day (*n* = 5). For interday precision and accuracy, LLOC and the three QCs were prepared five times and measured in duplicate on four separate days (*n* = 20). Absolute recovery was determined by comparing the mean peak areas of the ISZ and internal standard acquired after injection of the processed plasma samples with the peaks achieved by direct injection of the identical amount of compound diluted in the mobile phase (*n* = 5 for each concentration level).

### Effect of dilution and carryover.

The effect of dilution of plasma samples was validated with a 160.0-mg/liter spiked plasma sample diluted to the HQC level in plasma and analyzed five times. Carryover was tested by injecting two blank plasma samples immediately after five HQC ISZ injections, followed by evaluation of ISZ disappearance.

### Matrix effect and selectivity.

The matrix effect was evaluated by collecting blood from the same person in four different BD Vacutainer tubes (lithium heparin [LH], coagulation sodium citrate at 3.2% [CSC], EDTA [K2E], and clot activator for serum separation [serum]). The selectivity of the method was evaluated for interference of endogenous matrix components by analyzing six blank serum samples collected in clot activator tubes from different healthy human volunteers. For these human plasma and serum samples, intraday accuracy and precision values based on five replicates were calculated on the LQC and HQC samples as described above. Furthermore, selectivity for ISZ was evaluated together with a mixture of ITZ, OH-ITZ, PSZ, VRZ, and the internal standard obtained from the PCS and PCL I to III controls from ChromSystems as described above. The manufacturer’s manual includes a long list of immunosuppressants and antibacterial agents that do not cause interference when this TDM kit is used. The echinocandin caspofungin has also been tested by the manufacturer and shown to not be detectable with this kit at therapeutic levels. However, the manual mentions one example of a patient in intensive care who was administered clindamycin, fosamprenavir, pyrimethamine, piperacillin, sulfamethoxazole, tazobactam, and trimethoprim, and minor interferences were noticed in the VRZ range. For all of the compounds tested by the manufacturer, no interference was observed in the ITZ/ISZ range.

### Stability.

Stability of ISZ was investigated in human plasma (LQC and HQC) and evaluated after short-term and long-term storage. The stability at room temperature was examined by preparing and analyzing five replicates of the LQC and HQC plasma samples after 24 h and 7 days of incubation. Similarly, stability was examined after 48 h and 60 days of incubation at −20°C as well as after 24 h at 20°C in a solution also containing the internal standard. The effects of repeated freezing and thawing on LQC and HQC plasma samples were studied after three freeze-thaw cycles. Each freeze-thaw cycle consisted of a minimum of 12 h frozen at −20°C followed by a complete thaw at room temperature. Samples were prepared and analyzed after the third freeze-thaw cycle. Finally, postpreparative stability was determined for processed LQC and HQC plasma samples left in the autosampler (20°C) for 24 h (autosampler stability). The concentration after each storage period was considered stable if the mean concentration of each level was within ±15% of the nominal concentration.
