# Supplementary material for: Implementation of Isavuconazole in a Fluorescence-Based High-Performance Liquid Chromatography Kit Allowing Simultaneous Detection of All Four Currently Licensed Mold-Active Triazoles
Source: mSphere. 2017 May 10;2(3):e00098-17. doi: 10.1128/mSphere.00098-17 (PMC5425791; doi:10.1128/mSphere.00098-17)
Supplement: FIG S1 [file sph003172283sf2.pdf]

Supplemental Figure

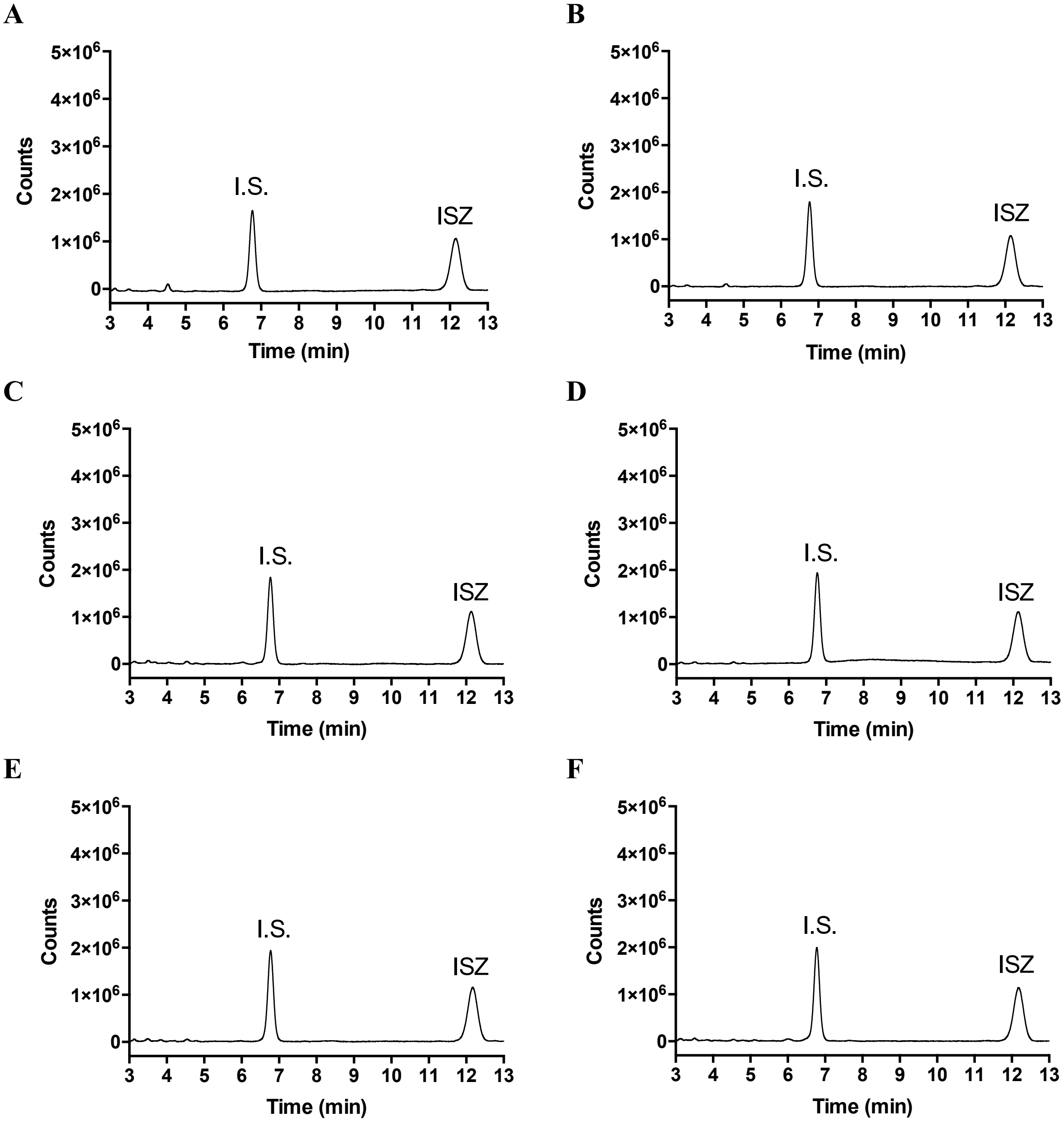

Fig S1. Chromatogram of serum samples spiked with ISZ from six healthy volunteers (A-F). The concentration of ISZ is 0.3 mg/L and is prepared together with the I.S supplied with the TDM kit.
