# Supplementary material for: Implementation of Isavuconazole in a Fluorescence-Based High-Performance Liquid Chromatography Kit Allowing Simultaneous Detection of All Four Currently Licensed Mold-Active Triazoles
Source: mSphere. 2017 May 10;2(3):e00098-17. doi: 10.1128/mSphere.00098-17 (PMC5425791; doi:10.1128/mSphere.00098-17)
Supplement: TABLE S1 [file sph003172283st1.pdf]

**Sample concentration (mg/L)** (Serum)	**Concentration found (mean mg/L ± S.D.)**	**Accuracy[1] (%)**	**Precision[2] (% R.S.D.)**
Person A			
LQC (0.3. n=5)	0.305±0.029	101.6	9.53
HQC (20.0, n=5)	21.037±0.368	105.2	1.07
Person B			
LQC (0.3, n=5)	0.285±0.005	95.0	1.92
HQC (20.0, n=5)	21.277±1.071	106.4	5.09
Person C			
LQC (0.3, n=5)	0.275±0.014	91.6	5.14
HQC (20.0, n=5)	20.959±1.191	104.8	5.68
Person D			
LQC (0.3, n=5)	0.270±0.007	90.0	2.44
HQC (20.0, n=5)	21.988±0.869	109.9	3.95
Person E			
LQC (0.3, n=5)	0.290±0.011	96.8	3.72
HQC (20.0, n=5)	22.074±1.129	111.0	5.09
Person F			
LQC (0.3, n=5)	0.267±0.004	89.1	1.40
HQC (20.0, n=5)	22.453±0.216	112.3	0.96

[1]Accuracy is percent of the nominal value.
[2]Precision is expressed as % relative standard deviation (R.S.D.) of each calculated concentration.
